# Hadrontherapy for Thymic Epithelial Tumors: Implementation in Clinical Practice

**DOI:** 10.3389/fonc.2021.738320

**Published:** 2021-10-11

**Authors:** Pierre Loap, Viviana Vitolo, Amelia Barcellini, Ludovic De Marzi, Alfredo Mirandola, Maria Rosaria Fiore, Barbara Vischioni, Barbara Alicja Jereczek-Fossa, Nicolas Girard, Youlia Kirova, Ester Orlandi

**Affiliations:** ^1^ Department of Radiation Oncology, Institut Curie, Paris, France; ^2^ Radiation Oncology Clinical Department, National Center for Oncological Hadrontherapy (CNAO), Pavia, Italy; ^3^ Institut Curie, Paris Sciences & Lettres (PSL) Research University, University Paris Saclay, laboratoire d’Imagerie Translationnelle en Oncologie, Institut National de la Santé et de la Recherche Médicale (INSERM LITO), Orsay, France; ^4^ Department of Oncology and Hemato-oncology, University of Milan, Milan, Italy; ^5^ Division of Radiotherapy, Istituto Europeo di Oncologia (IEO) European Institute of Oncology Istituti di Ricovero e Cura a Carattere Scientifico (IRCCS), Milan, Italy; ^6^ Institut du Thorax Curie Montsouris, Paris, France; ^7^ Department of Medical Oncology, Institut Curie, Paris, France; ^8^ University Paris Saint-Quentin, Versailles, France

**Keywords:** thymoma, thymic carcinoma, proton therapy, carbon ion therapy, hadrontherapy

## Abstract

Radiation therapy is part of recommendations in the adjuvant settings for advanced stage or as exclusive treatment in unresectable thymic epithelial tumors (TETs). However, first-generation techniques delivered substantial radiation doses to critical organs at risk (OARs), such as the heart or the lungs, resulting in noticeable radiation-induced toxicity. Treatment techniques have significantly evolved for TET irradiation, and modern techniques efficiently spare normal surrounding tissues without negative impact on tumor coverage and consequently local control or patient survival. Considering its dosimetric advantages, hadrontherapy (which includes proton therapy and carbon ion therapy) has proved to be worthwhile for TET irradiation in particular for challenging clinical situations such as cardiac tumoral involvement. However, clinical experience for hadrontherapy is still limited and mainly relies on small-size proton therapy studies. This critical review aims to analyze the current status of hadrontherapy for TET irradiation to implement it at a larger scale.

## 1 Introduction

Thymic epithelial tumors (TETs) represent a noticeable heterogeneous group of rare thoracic malignancies, including thymomas and thymic carcinomas, with an estimated incidence of 1.3 and 3.2 cases per million person-years (1). When feasible, surgery is the gold standard, but radiation therapy (RT) plays an important role in radical and adjuvant settings. In particular, RT is part of the recommendations for locally advanced (stage III–IV) TETs after surgery, especially in cases of thymic carcinomas or positive margins, or as a radical treatment for unresectable patients. For R0-resected localized TETs, adjuvant RT is recommended in cases of thymic carcinoma histology, since it significantly increases recurrence-free survival and overall survival; the clinical benefit or RT seems however inexistent for completely resected stage I thymomas and is debatable in other stages (2). First-generation RT techniques relied on two-dimensional (2-D) planning, which exposed critical organs at risk (OARs) to substantial doses (such as the heart or the lungs) and was consequently associated with significant toxicity (3). The technical evolution of RT allows to better spare OARs without altering the tumor coverage and consequently the local control. Such breakthroughs included intensity-modulated RT (IMRT) and respiratory control techniques such as respiratory gating and deep-inspiration breath hold (DIBH). Nevertheless, despite these advances, some clinical situations (i.e., pericardial or myocardial tumoral involvement) are still challenging even with highly conformal IMRT. Particle beams of protons or carbon ions are able to deliver most of their energy towards the end of the particle range resulting from an increased linear energy transfer (LET) before particle rest, in the well-known Bragg peak (4). Consequently, distant-to-target dose deposition is substantially reduced compared with conventional photon RT technique. Proton RT has been recently considered for TET irradiation in patients with significant baseline cardiac risk factors or with cardiac tumoral involvement. However, clinical experience of hadrontherapy for TET irradiation is still limited. The purpose of this review is to provide a contextualized analysis of the status of hadrontherapy in TET management.

## 2 Expectations on Hadrontherapy for Thymic Epithelial Tumors

### 2.1 Clinical Considerations

#### 2.1.1 Cardiotoxicity Risk Reduction

The cause-specific mortality analysis on a retrospective series of the SEER database reported no significant difference (p = 0.68) in cardiac mortality rate between the TET patients who had received RT (14.3%) and those who had not (12.9%), with a non-statistically significant difference in terms of cardiac death incidence between the two groups (3.4% vs. 5.9% at 6 years and 17.4% vs. 11.8% at 24 years for irradiated and non-irradiated patients, p = 0.85) (5). This delayed increase of cardiac death might be related to the late toxicity, for which long-term follow-up is needed. The potential benefit of hadrontherapy for late cardiac adverse event reduction is based on the improved cardiac sparing capacity of particle beams, compared with photon RT techniques. In the *in silico* study by Vogel et al., proton beam plans delivered in a cohort of 22 TET patients were reoptimized with an IMRT approach showing a significant reduction in dose to the heart and left ventricle (6). Based on a linear relationship between MCE and mean heart dose (MHD) (7), major cardiac event (MCE) risk was significantly lower with proton beam therapy (PBT) compared with IMRT (74% vs. 135%, p = 0.04) (6). Using a linear relationship between MCE and MHD developed for Hodgkin’s lymphoma (8), Franceschini et al. (9) evidenced that PBT would significantly reduce congestive heart failure incidence when compared with volumetric modulated arc therapy (VMAT) in an adjuvant setting, with a relative risk (RR) of 1.3 for PBT and of 1.6 for VMAT.

#### 2.1.2 Pulmonary Toxicity Risk Reduction

Moiseenko et al. (3) proposed a normal tissue complication probability (NTCP) model, based on the Lyman formalism, from a cohort of 55 thymoma patients treated with photon RT. In this study, the mean lung dose (MLD) significantly correlated with symptomatic acute pneumonitis and late lung fibrosis. It should be stressed that patients included in this study were treated with outdated techniques, including ^60^Co 2-D irradiation. Whether this NTCP model is valid for IMRT and hadrontherapy is an open question but justifies lowering as much as possible MLD during TET irradiation. Expectedly, Swisher-McClure et al. (10) demonstrated in a limited-size dosimetric study that PBT was associated with a significant dosimetric reduction of lung dosimetric parameters (including MLD, V20, and V5) in an adjuvant setting. In addition, using NTCP models, Lidestahl et al. (11) demonstrated that the risk of pneumonitis would be significantly lower with PBT than IMRT or 3D-RT (respectively, 2.5%, 10.8%, and 9.1%).

#### 2.1.3 Toxicity to Other Organs at Risk

PBT has proved to be worthwhile in decrease radiation-induced esophagitis (4.3% with PBT vs. 5.8% with IMRT) and myelopathy (0% with PBT vs. 0.4% with 3D-RT) (11); however, these reductions were of limited clinical amplitude. In the mono-institutional *in silico* experience by Haefner et al. (12), particle therapy (carbon ion radiation therapy (CIRT) and PBT) gives lower doses to the heart, lungs, breast, esophagus, and spinal cord, than did the conventional RT approach (VMAT, helical tomotherapy (HT), and 3D-RT). Moreover, among photon beam RT, HT was associated with substantial low-dose exposure to the lungs, breasts, and heart. While the effects of low-dose exposure on carcinogenesis are subject to notable debate, lowering cumulative radiation dose to OARs may result in fewer secondary cancers (13). Franceschini et al. (9) found a substantial risk reduction of secondary cancer induction with PBT compared with VMAT based on the Schneider model (14): notable decreases in excess absolute risk (EAR) of esophagus cancer (EAR of 3.6 vs. 1.0–1.2/10,000 patient-years), breast cancer (EAR of 17.4 vs. 5.7–6.1/10,000 patient-years), and lung cancer (EAR of 24.8 vs. 8.1–8.7/10,000 patient-years) were observed. Similarly, Vogel et al. (15) estimated that five excess secondary malignancies per 100 patients would be avoided by treating TET patients with PBT instead of IMRT.

### 2.2 Biological Considerations

#### 2.2.1 Immunomodulation of the Tumor Microenvironment

TETs are associated with one of the lowest tumor mutation burden (TMB) among all adult cancers as well as a notable intratumoral heterogeneity concerning PD-L1 and PD-1 expression (16). Indeed, high PD-1 expression is associated with a lower tumor grade, contrary to PD-L1 expression, which does not correlate with tumor grade, since PD-L1 expression is constitutive of TETs (16). TET patients present a notable increase in extrathymic cancers (17), and there has been increased suspicion of immune disturbance leading to defective cancer immunosurveillance. An additional argument for immune disturbance is the frequency of autoimmune diseases, such as myasthenia gravis. Abscopal effects after RT for TETs have been reported, suggesting possible RT immunomodulation in the microenvironment (18, 19). Notably, one abscopal case report followed the use of CyberKnife stereotactic radiotherapy (20). In this context, heavy-ion RT is of particular interest. Spina et al. (21) and Simoniello et al. (22) unambiguously demonstrated that CIRT could efficiently induce pro-inflammatory cytokines, while sparing circulating lymphocytes, which could polarize the tumor microenvironment into an antitumor one. For their physical selectivity, fewer chromosomal aberrations were described in patients treated with CIRT than with photon beam RT (23–25), leading to a higher number of available immune cells that might be recruited for the immune response after cancer (26). Moreover, the radiobiological hallmarks of CIRT can lead to a production of double-stranded DNA (dsDNA) scraps that have been proved to enhance the immune response (26). Even the above results are promising but still inconclusive; several strategies are under study to induce an abscopal effect; and considering their characteristics, TET might be a suitable study target.

#### 2.2.2 Hypoxia

In addition, TET represents a highly heterogeneous cancer group at the molecular level. Thymomas are associated with a homogeneous ^18^F-FDG uptake, and more aggressive thymic carcinomas are characterized by a heterogeneous one (27). Kaira et al. (28) reported a ^18^F-FDG uptake correlation with the upregulation of hypoxia-inducible factor (HIF)-1α, a transcription factor that plays a key role in hypoxic adaptation of neoplastic cells. Overexpression of HIF-1α is related to aggressiveness and scant prognosis (29). High expression level of hypoxia-related genes was reported in TET (30). In particular, carbonic anhydrase 9 (CA9) level was associated with Masaoka stage, World Health Organization classification, and relapse-free survival in the adjuvant setting (30). CA9 was found to be expressed in 81% of thymic carcinomas and 21% of all TETs (30). In addition, preclinical data on mice models demonstrated the existence of quiescent radioresistant epithelial progenitors (31), which might exist as well in humans. In this context, hadrontherapy might be beneficial in cases of such heterogeneous hypoxic tumors, due to the reduced effect of tissue oxygenation on antitumor efficacy of particle beams.

## 3 Current Experience of Hadrontherapy for Thymic Epithelial Tumors

While particle beam therapy demonstrated a theoretical dosimetric benefit for TET irradiation, the rarity of this tumor as well as the smaller number of available particle facilities might explain the paucity of available clinical data. Most of the evidence relies on PBT.

### 3.1 Clinical Evidence

Current clinical experience of hadrontherapy for TETs is summarized in **Table 1**.

**Table 1:** Current experience on hadrontherapy for thymic epithelial tumors.

Study	Size	Particle	Technique	Radiation therapy setting	Dose (RBE)	Follow-up	Efficacy	Tolerance
Figura et al. (32)	1 pt.	Proton	*NA*	Adjuvant	50.4 + 10.8 Gy	*NA*	*NA*	*NA*
Sugawara et al. (33)	1 pt.	Proton	DS. Respiratory gating	Definitive (cardiac invasion)	74 Gy	*NA*	*NA*	*NA*
Parikh et al. (34)	4 pts.	Proton	US	Adjuvant	57 Gy [50.4–66.6 Gy]	5.5 months	No relapse	One grade 2 dermatitis. No grade ≥3 toxicity
Vogel et al. (35)	27 pts.	Proton	DS. Respiratory gating	Adjuvant (63%), definitive (22%) and relapse (15%)	61.2 Gy [50.4–70.2 Gy]	2.0 years	2-year local control: 100%.	Grade 2 dermatitis (37%), esophagitis (7%), and pneumonitis (4%). No grade ≥3 toxicity
							3-year regional control: 96%.	
							3-year distant control: 74%. 3-year overall survival: 94%	
Zhu et al. (36)	6 pts.	Proton	DS. Respiratory gating	Adjuvant (83%), definitive (17%)	60 Gy [54–74 Gy]	2.6 years	Local control at 2.6 years: 100%.	Grade 2 dermatitis (83%), grade 2 esophagitis (17%). No grade ≥3 toxicity
							2 out-of-field recurrences	
Hayashi et al. (37)	1 pt.	Carbon ion	Respiratory gating	Metastatic (lung)	52.8 Gy (12 fractions)	*NA*	*NA*	*NA*
Mercado et al. (38)	22 pts.	Proton	DS, US, and PBS. Respiratory gating	Adjuvant (91%), definitive (9%)	54 Gy [45–70 Gy]	13 months	5 relapses (including 1 local relapse)	Grade 2 dermatitis (37%), cough (13%) and esophagitis (10%). No grade ≥3 toxicity
Fukai et al. (39)	1 pt.	Proton	*NA*	Definitive (progressive residual intramyocardial lesion)	50 Gy	*NA*	*NA*	*NA*
Loap et al. (40)	1 pt.	Proton	DS. DIBH	Definitive (primitive lesion + pericardial nodules)	60 Gy	*NA*	*NA*	*NA*
McGunigal et al. (41)	7 pts	Proton	PBS. Respiratory gating	Adjuvant	54 Gy	21 months	No relapse	Grade 2 dermatitis (29%). No grade ≥3 toxicity)

DS, double scattering; PBS, pencil beam scanning; US, uniform scanning; NA, non-assessable; Pt, patient; Gy, Gray.

#### 3.1.1 Hadrontherapy

The first case report of PBT for TETs has been reported by Figura et al. (32) in an adjuvant context: a 23-year-old female patient was treated for a stage III thymoma with initial surgery with positive margins; considering her young age and due to the risk of long-term complication of thoracic RT based on the initial IMRT plan evaluation, it was ultimately decided to deliver adjuvant RT with PBT to a total dose of 50.4 Gy. No tolerance data were reported in this first case report. Parikh et al. (34) demonstrated on four patients treated in an adjuvant context an excellent toxicity profile without any grade ≥3 adverse events. Vogel et al. (35) described the efficacy outcomes of PBT on a cohort of 27 TET patients (85% thymoma and 15% of thymic carcinomas) treated for 63% in an adjuvant context, for 22% in a definitive context, and 15% in recurrent disease. The 2-year local control was 100%; 3-year regional control was 96%, 3-year distant control was 74%, and 3-year overall survival was 94%. PBT was well-tolerated without grade ≥3 toxicity. Zhu et al. (36) described similar outcomes on a small cohort of six patients in terms of toxicities (no grade 3) and local control (after a median follow-up of 2.6 years, two out-of-field recurrences were observed). Compared with IMRT, MHD was reduced by 36.5%, MLD by 33.5%, and mean dose to the esophagus by 60%. Mercado et al. (38) confirmed on a cohort of 22 patients the good tolerance profile of PBT for TETs where the most frequent adverse event was a grade 2 dermatitis, occurring in 37% of patients. With a median follow-up of 13 months, there were five relapses, including one local. Finally, McGunigal et al. (41) evaluated recent Monte Carlo dose calculation algorithms for pencil beam scanning (PBS)-PBT on a cohort of seven patients in an adjuvant setting with no relapse after 21 months. Considering the above reported results, with all their limitations (small simple size, retrospective data, and lack of data on follow-up), PBT for TET irradiation seems to be well tolerated, without any grade ≥3 toxicity reported to date and is associated with a promising local control. Longer follow-up and a prospective series are however necessary to confirm these preliminary results in terms of tolerance and efficacy. The ongoing PROTHYM single-arm phase-2 trial (NCT04822077) intends to recruit 40 patients to precise cardiac and pulmonary toxicities and 5-year local control with PBT for TET irradiation.

CIRT has been seldomly used for TET irradiation. In the series of Hayashi et al. (37), one of the 95 patients treated with CIRT for lung metastases has a TET lung localization. The patient underwent up to a total dose of 52.8 Gy of relative biological effectiveness (RBE) in 12 fractions without concurrent chemotherapy. No further specific data are available.

#### 3.1.2 Specific Clinical Situations

The tumoral involvement of cardiac substructures in advanced-stage TETs is a challenge for radiation oncologist considering the significant cardiotoxicity risk. Hadrontherapy might be of interest to limit radiation exposure to unaffected cardiac substructure as described also in non-oncological settings (42). With regards non-metastatic TETs, Sugawara et al. (33) reported the use of PBT to treat a large cardiac-invading TET in a definitive setting, Loap et al. (40) described the PBT treatment of anterior pericardial nodules of a stage IVB TET, and Fukai et al. (39) irradiated an evolutive intramyocardial post-surgery residue. These challenging situations, where planned target volumes include part of the heart, might be associated with a limited control (40) and pose specific technical challenges. While respiratory motion control strategies relying on gating or DIBH techniques are widespread, cardiac movement is challenging to take simultaneously into account. To this end, dual ECG-respiratory gating techniques have been proposed (43).

### 3.2 Treatment Considerations

#### 3.2.1 Proton Therapy Technique

The optimal respiratory control technique for TET PBT is still undefined yet. Most clinical experience on PBT used 4D-CT gating systems (35, 36, 41), which is particularly convenient for patients that may have limited breathing capacities resulting from advanced-stage disease. DIBH, possibly controlled with spirometers, is an alternative that may limit range uncertainty and reduce target volumes (40). Dosimetric comparison studies between DIBH and FB with respiratory gating are equivocal. Rechner et al. (44) found on seven TET patients that DIBH would be associated with a dose reduction to the heart and to the lungs; on the other hand, Fracchiolla et al. (45), focusing on PBS-PBT with respiratory gating, did not find any significant interplay effect due to breathing on TET plans. In addition, a comparison between the two main PBT delivery modalities, PBS and double scattering (DS), has not been conducted yet. Loap et al. (40) estimated that PBS would lower skin dose compared with DS. However, in daily practice, PBS tends to become the only delivery modality available in new particle treatment centers, partly due to its increased conformity characteristics. It should be kept in mind, however, that PBS may have a greater interplay effect than DS, which could justify rescanning or tracking techniques (46), and a larger lateral penumbra (47). The lateral penumbra corresponds to the lateral dose fall-off, depends on the PBT system design and on setup parameters, and is an important point to consider for the dosimetric sparing of the OARs adjacent to the proton beams.

#### 3.2.2 Treatment Planning

There are small variations in published treatment volumes for TET PBT. Zhu et al. (36) defined gross target volume (GTV) as radiological disease at diagnosis, including ^18^F-FDG imagery modality. Clinical target volume (CTV) was defined as the GTV (in case of adjuvant PBT) or as the postoperative bed (in case of definitive PBT) with a margin of 5 mm. An internal target volume (ITV) was defined on the 10 phases of a 4D-CT simulation scanner. Planning target volume (PTV) was defined as ITV with a margin of 5 mm. On the other hand, Vogel et al. (35) contoured the GTV on multiple phases of a 4D-CT scan; an ITV was defined as the fusion of the GTV with a margin of 8 mm. PTV was defined as ITV and a margin of 5 mm. In addition, treatment planning algorithms are also evolving for TET PBT: evaluation of robust optimization planning algorithms has been evaluated for TET proton therapy by Franceschini et al. (9), while McGunigal et al. (41) demonstrated that Monte Carlo algorithms might lead to more realist dose calculations compared with standard pencil beam calculation algorithms.

#### 3.2.3 Practical Recommendations for Thymic Epithelial Tumor Hadrontherapy

**Figure 1:**
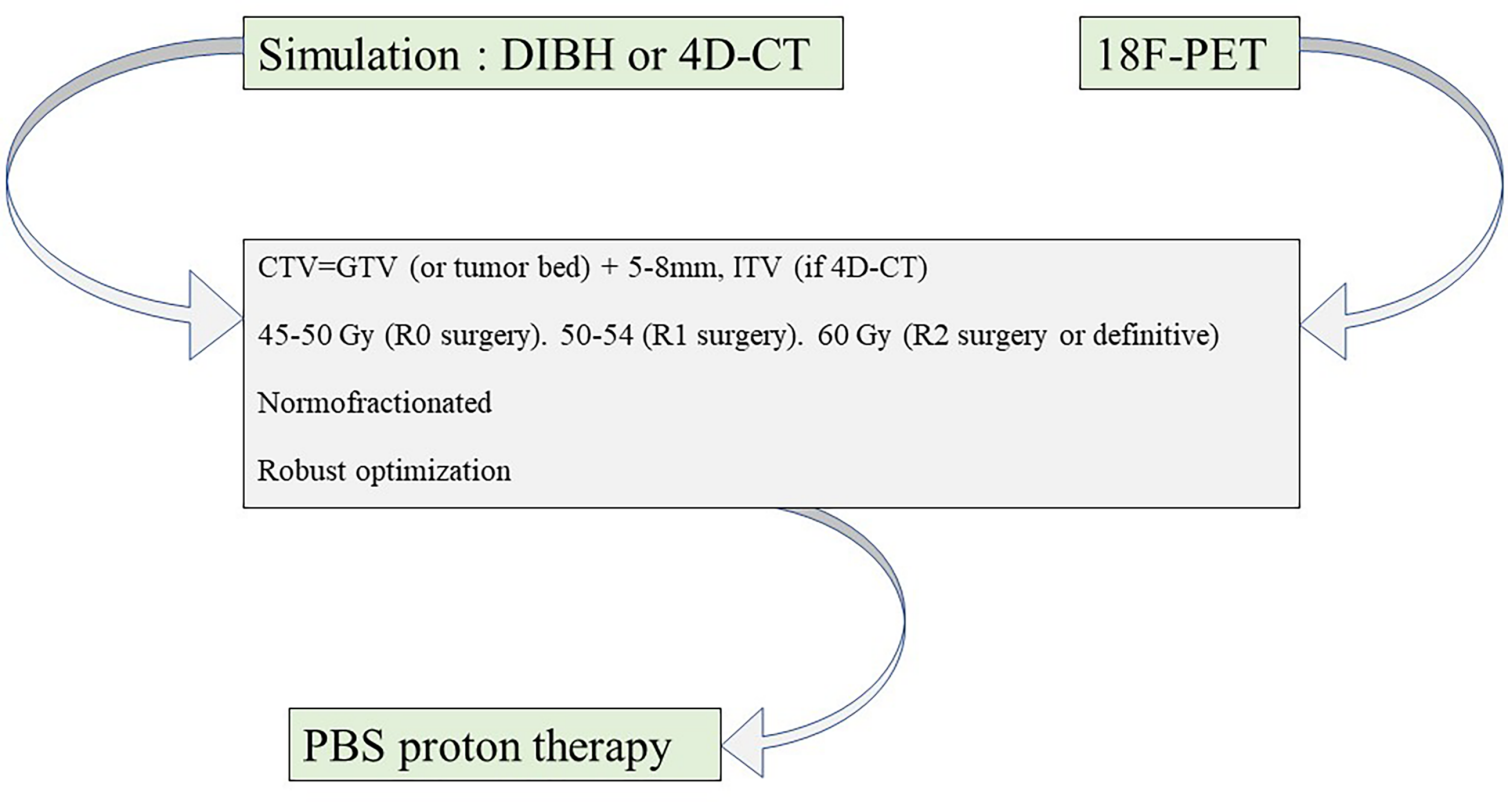
Proton therapy planning for thymic epithelial tumor irradiation. CT, computed tomography; ^18^F-PET, fluorodeoxyglucose F18 positron emission tomography; DIBH, deep-inspiration breath hold; PBS, pencil beam scanning; GTV, gross target volume; ITV, internal target volume; CTV, clinical target volume.

Practical propositions for modern hadrontherapy for TETs are summarized in **Table 2** and in **Figure 1**. Hadrontherapy may be proposed for selected TET patients, in both postoperative or definitive settings, in case of significant cardiotoxicity risk, such as cardiac tumoral involvement or patient-specific clinical considerations (cardiovascular history and risk factors, and baseline lung disease). A ^18^F-FDG PET may be realized to better define GTV, in addition to the radiomic value of this imagery modality (27). Patient simulation should take into account the range uncertainties of particle beams, and a respiratory control should be included, either 4D-CT or DIBH. DIBH could be spirometer-controlled, when possible (48). The volume definition and contouring rely on published guidelines. Most publications on TET PBT defined CTV as the GTV or the postoperative bed with a margin of 5–8 mm; in addition, according to European Society for Medical Oncology (ESMO) guidelines (2), the whole thymic space, the tumor and its extension, and the anterior and superior-middle mediastina should be included in the CTV. An ITV is necessary in case of 4D-CT acquisition but not in case of DIBH. Several studies are ongoing to further define reproducible target volumes, such as the ongoing RADIORYTHMIC trial (NCT04731610) (49). For PBT, the prescribed dose should be the same as for photon RT, following published guidelines. ESMO guidelines recommend 45–50 Gy after R0 resection, 50–54 Gy after R1 resection, and 60 Gy after R2 resection or in a definitive setting. PBS might be preferred over DS due to its increased conformity. Rescanning should be considered to mitigate interplay effect in case of 4D-CT planning. Robust optimization should be recommended. ECG gating, when possible, should be considered in case of target volumes next to the heart or in case of cardiac tumoral involvement. 4D-MRI and four-dimensional restricted robust optimization should be considered especially for CIRT treatment (50).

**Table 2:** Practical propositions for hadron therapy for thymic epithelial tumor irradiation.

Treatment planning phase	Proposition	Remark
Initial imaging	^18^F-FDG PET	Target delineation.
		Radiomic prognosis.
Patient simulation	4D-CT scans or DIBH	DIBH may be spirometer-controlled
Delineation and dose	According to guidelines for photon RT	CTV = GTV (or tumor bed) + 5–8 mm.
		Thymic loge, tumor expansion, and anterior upper-middle mediastina to be included in the CTV according to ESMO guidelines.
		ITV to be delineated if 4D-CT acquisition.
		PTV margins according to local referential (usually, +5 mm)
		Dose: 45–50 Gy (R0 surgery). 50–54 (R1 surgery). 60 Gy (R2 surgery or definitive)
Particle	Proton therapy should be preferred	Limited experience for CIRT (metastatic sites only)
Particle delivery modality	For proton therapy: PBS.	For CIRT: PBS
	DS possible when PBS is not available	
Fractionation	For proton therapy: 1.8-2.0 Gy/fraction	For CIRT (metastatic site): 4.4 Gy/fraction, 12 fractions
Dose calculation	Monte Carlo algorithms	
Planning	Robust planning algorithms	

^18^F-PET, fluorodeoxyglucose F18 positron emission tomography; CIRT, carbon ion radiation therapy; DIBH, deep-inspiration breath hold; CT, computed tomography; PBS, pencil beam scanning; DS, double scattering; GTV, gross target volume; ITV, internal target volume; CTV, clinical target volume.

## 4 Discussion

Hadrontherapy has been proposed for TET treatment based on dosimetric considerations from which a reduction in late adverse events is expected (in particular, for cardiac and lung toxicities). Short-term follow-up of current studies demonstrates an excellent tolerance profile to this date. However, the frequency of autoimmune diseases in patients with TETs should be kept in mind. It might be explained by the release of immature auto-reactive T cells that have not undergone negative selection, a physiologic function of the thymus (51). The pro-inflammatory signals induced by particle beam (especially in case of CIRT) may lead to an immunological activation of auto-reactive T-lymphocyte clones. Jakopovic et al. (52) demonstrated that TETs were associated with increased activation of auto-reactive T-lymphocyte clones under immunotherapy treatment compared with other cancer types. Lymphocytes are particularly sensitive to ionizing radiations and die off at a low dose level. Multiple studies have demonstrated a significant sparing of circulating lymphocytes with hadrontherapy compared with photon RT (53, 54), which is one of its theoretical advantages. However, the impact on antitumor immunity and on clinical outcome is still to be precisely evaluated.

A significant proportion of myocarditis-type adverse events were reported in TET patients undergoing immunotherapy treatment, compared with other cancer types (55). Future trials potentially evaluating a potential combination of immunotherapy and hadrontherapy should be consequently done cautiously. Similarly, a potential immune-activating abscopal effect on auto-reactive T-lymphocyte clones against cardiac antigens might act synergically with direct radiation-induced damage on cardiac substructures: the localization of critical cardiac substructures, such as the left anterior descending coronary artery (LADCA), the right coronary artery, or the left ventricle is localized close or at the contact of the target volumes. In this situation, these OARs are localized in a zone where the RBE value is uncertain and where the classic RBE value of 1.1 might not be valid. Variable RBE planning algorithms might consequently be considered (56). In addition, cardiac movement is usually not taken into account during treatment planning since ECG-gated treatments are not generalized yet. However, proton beams are extremely sensitive to range uncertainties, and cardiac intrinsic movement might consequently lead to over-dosing on coronary arteries. Use of planned OAR volumes for the LADCA (57) or specific surrogate OAR (58), associated with robust planning algorithms, might reduce this potential cardiotoxicity risk.

To this date, RT is recommended for inoperable patients, in the adjuvant setting after surgery in cases of R1–R2 residue and possibly after R0 surgery (depending on stage and histology); RT can be combined with chemotherapy (2). Surgical techniques are rapidly evolving (59), and the indications for adjuvant RT might consequently evolve. In addition, clear selection criteria for TET hadrontherapy (over photon RT) are still to be precisely defined, but Glimelius et al. (60) grossly estimated that 50% of TET patients could benefit from proton therapy to reduce acute and long-term side effects. The location of the target volumes in relation to the OARs is the prime determinant of radiation-induced toxicities. It should be noted that in the adjuvant setting, the target volumes are usually located above most cardiac substructures (including the coronary arteries); consequently, the dosimetric benefit of hadrontherapy may not be clinically significant in this situation, since high doses to cardiac substructures should theoretically be limited regardless of the RT technique. However, in the definitive setting, when R0 tumor resection is unrealistic due to an extensive disease extent or when the tumor abuts the heart, hadrontherapy is expected to substantially spare cardiac substructures compared with photon RT.

There is an unequal access to hadrontherapy facilities around the world. In Europe, the European Particle Therapy Network has been created to ease international cooperation and to enhance clinical research on hadrontherapy (61), which is of prime importance for rare tumors like TETs. The development of large registries can increase the evidence level of hadrontherapy. Nevertheless, reimbursement issues exist for tumor types with low evidence levels for hadrontherapy such as TETs, which is currently not widely recognized as a hadrontherapy indication (62). No cost-effectiveness analyses or NTCP-model-based evaluations (63) have been conducted to this date. Finally, adjuvant irradiation, which represents most of TET hadrontherapy indication, might not be prioritized over definitive treatment of aggressive or in-place tumors at the level of a given typical hadrontherapy center with limited treatment resources (64).

In conclusion, hadrontherapy for TET irradiation has the potential to significantly reduce radiation exposure to several OARs, including cardiac substructures, which should substantially reduce late radiation-induced toxicities and secondary cancer risk. While hadrontherapy could be useful in the case of complex clinical presentation with cardiac tumoral involvement, its implementation in clinical practice is facing technical and societal challenges, and its clinical benefit is difficult to evaluate in practice due to limited available data and short follow-up. Large registries might help to increase the evidence of hadrontherapy in this indication.

## Author Contributions

Conceptualization: PL, YK, and EO. Methodology: PL, YK, and EO. Writing: PL. Review and editing: PL, LM, BJ-F, AM, AB, VV, NG, MF, BV, YK, and EO. Supervision: YK and EO. YK and EO contributed equally to the work. All authors contributed to the article and approved the submitted version.

## Conflict of Interest

The authors declare that the research was conducted in the absence of any commercial or financial relationships that could be construed as a potential conflict of interest.

## Publisher’s Note

All claims expressed in this article are solely those of the authors and do not necessarily represent those of their affiliated organizations, or those of the publisher, the editors and the reviewers. Any product that may be evaluated in this article, or claim that may be made by its manufacturer, is not guaranteed or endorsed by the publisher.

## Abbreviations

CIRT, carbon ion radiation therapy; DIBH, deep-inspiration breath hold; DS, double scattering; IMRT, intensity-modulated radiation therapy; IMPT, intensity-modulated proton therapy; OAR, organ at risk; MCE, major cardiac event; MHD, mean heart dose; MLD, mean lung dose; NTCP, normal tissue complication probability; PBS, pencil beam scanning; PBT, proton beam therapy; TET, thymic epithelial tumor; VMAT, volumetric modulated arc therapy.
